# Reusable, polyethylene glycol-structured microfluidic channel for particle immunoassays

**DOI:** 10.1186/1754-1611-3-6

**Published:** 2009-04-28

**Authors:** Jin-Hee Han, Jeong-Yeol Yoon

**Affiliations:** 1 Department of Agricultural and Biosystems Engineering, The University of Arizona, Tucson, Arizona 85721-0038, USA

## Abstract

A microfluidic channel made entirely out of polyethylene glycol (PEG), not PEG coating to silicon or polydimethylsiloxane (PDMS) surface, was fabricated and tested for its reusability in particle immunoassays and passive protein fouling, at relatively high target concentrations (1 mg ml^-1^). The PEG devices were reusable up to ten times while the oxygen-plasma-treated polydimethyl siloxane (PDMS) device could be reused up to four times and plain PDMS were not reusable. Liquid was delivered spontaneously via capillary action and complicated bonding procedure was not necessary. The contact angle analysis revealed that the water contact angle on microchannel surface should be lower than ~60°, which are comparable to those on dried protein films, to be reusable for particle immunoassays and passive protein fouling.

## Background

Polyethylene glycol (PEG) surfaces have been recognized to resist protein fouling due to their hydrophilic nature (water contact angle = ~20°). The existence of oxygen in their backbone -(CH_2_CH_2_O-)_n _and a high degree of H_2_O/PEG structural organization [1] enable the reversal of binding before the adsorbed protein "flattens out" and denatures through forming multiple attachments to a surface [2]. This protein fouling is a key problem in performing biological assays in a microfluidic device [3,4]. Therefore, there have been several attempts to modify their surfaces, including silicon and polydimethylsiloxane (PDMS), with PEG. These modifications include passive adsorption [5], chemical vapour deposition (CVD) [6,7]. These efforts have proved unsuccessful due to their fabrication complexity [8] or poor long-term stability (some PEG coatings may eventually come off from microchannel surface upon rinsing [8]). Alternatively, PEG has been covalently added to microchannel surface through self-assembling PEG-terminated alkyl silane on silicon-based surfaces. This coating is also known as PEG-SAM (the latter represents self-assembled monolayer) [9,10]. The potential problems of PEG-SAM include: (1) difficulty of immobilizing certain bioreceptors (e.g. antibodies) within a microchannel (PEG repels those bioreceptors), (2) uneven coating to complicated structures such as cross junctions, view cells, and microvalves, and (3) time-consuming, complex process of making PEG-SAM on the covered microchannel [8,11]. Chemical grafting of PEG onto silicon or PDMS has been attempted [12,13], which provided better quality final film. However, this method require multiple, difficult-to-control processing steps to achieve a high quality [8,14]. In addition, the organic solvents required for PEG coating would swell the PDMS network [15]. All these complications originate from the fact that the PEG layer is added to the existing silicon or PDMS surface.

A better alternative is to fabricate a microfluidic channel made solely out of PEG. Kim et al. [16] has recently fabricated a microchannel comprised entirely of PEG by cross-linking it through exposure to ultraviolet (UV) radiation. Indeed, their device was successful in resisting protein fouling, but a few complications could be found in their work. They discussed how to prevent PEG swelling and how to make better bonding between mold-replica or microchannel-cover slip, indicating potential fabrication complications. We have actually duplicated their technology and found that PEG device was not suitable for repeated uses. Bonding of a PEG microchannel to either a glass cover slide or another PEG substrate was found to be difficult due to their surface roughness. Leaking was observed from the very first use and became worse upon repeated use. In addition, they did not expose their device to repeated washing conditions that is common in practical biological assays. As expected, they did not performed actual biological assays with their device.

In this work, we expanded the work of Kim et al. [16] by (1) eliminating the bonding procedure between the PEG substrate and a glass cover slide, (2) demonstrating simpler liquid delivery via capillary action (Kim et al. used a micropump), and (3) performing actual biological assays in a repeatable manner at relatively high protein concentrations (Kim et al. tested 20–50 μg ml^-1^; we tested ~1 mg ml^-1^). Particle immunoassays for mouse immunoglobulin G (mIgG) were repeated at very high concentrations such that the reusability times could be estimated for the PEG microfluidic channel. Fluorescein-labelled bovine serum albumin (BSA) was also tested to further evaluate passive protein fouling.

## Results and discussion

### Simplified assembly/use of a PEG microchannel

**Figure 1:**
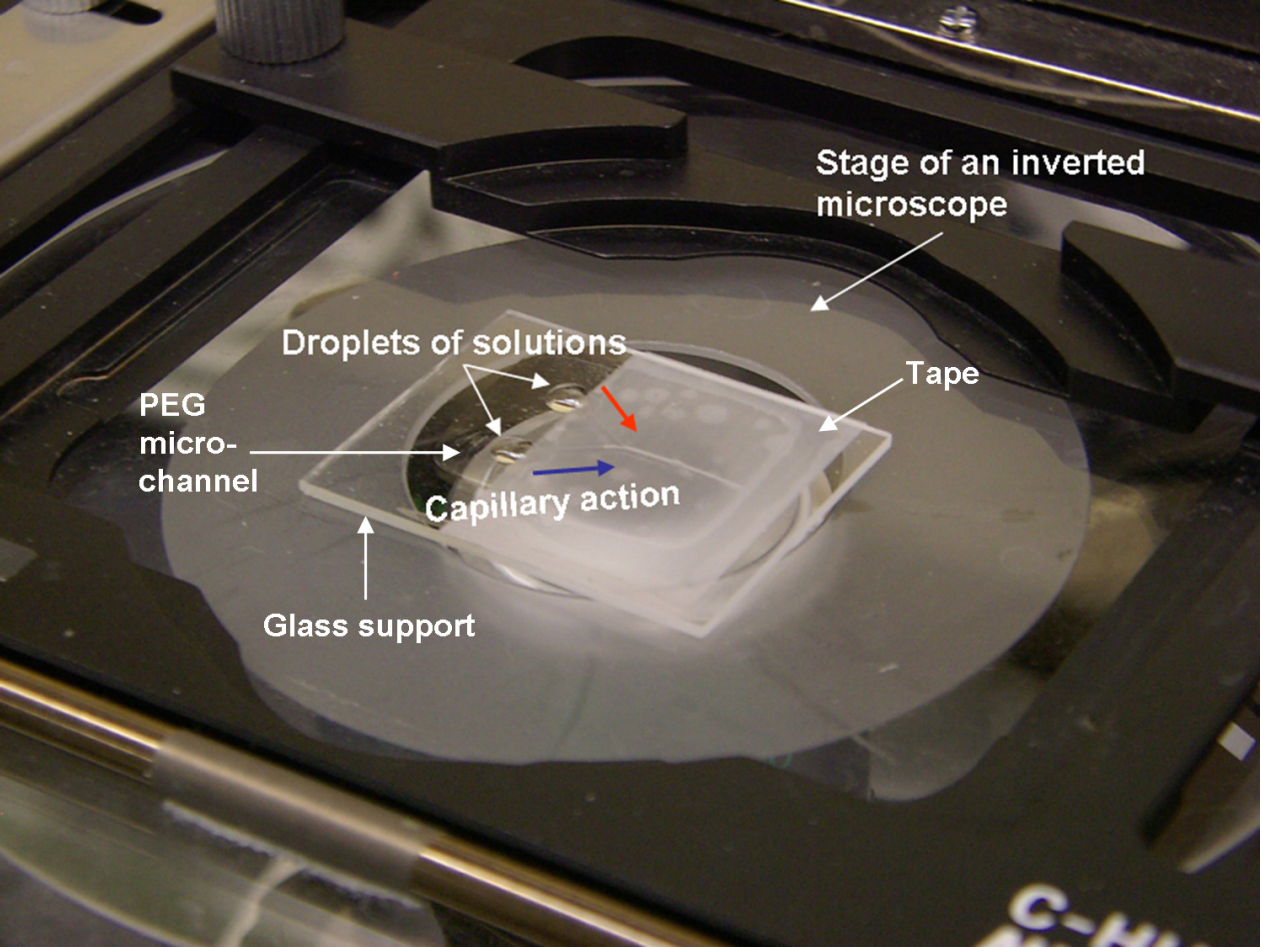
**Liquid droplets are delivered into microchannels via capillary action**. The inner microchannels are monitored with an inverted microscope. Glass support is 32 mm × 25 mm, while PEG microchannel area is 17 mm × 12 mm.

A syringe pump was required to deliver liquids into a PDMS microfluidic channel since its surface was hydrophobic. However, PEG microchannel did not require such pumping since its surface was hydrophilic and liquids could be delivered by capillary action (Fig. 1). The rigidity and surface roughness of cross-linked PEG substrate made bonding to a cover slide difficult, leading to liquid leaking. As addressed in Methods section, we used an adhesive tape as a cover slide, and were able to resolve both cover bonding and liquid leaking issues without any complicated bonding procedure.

### Reusability as observed with microscopic images

**Figure 2:**
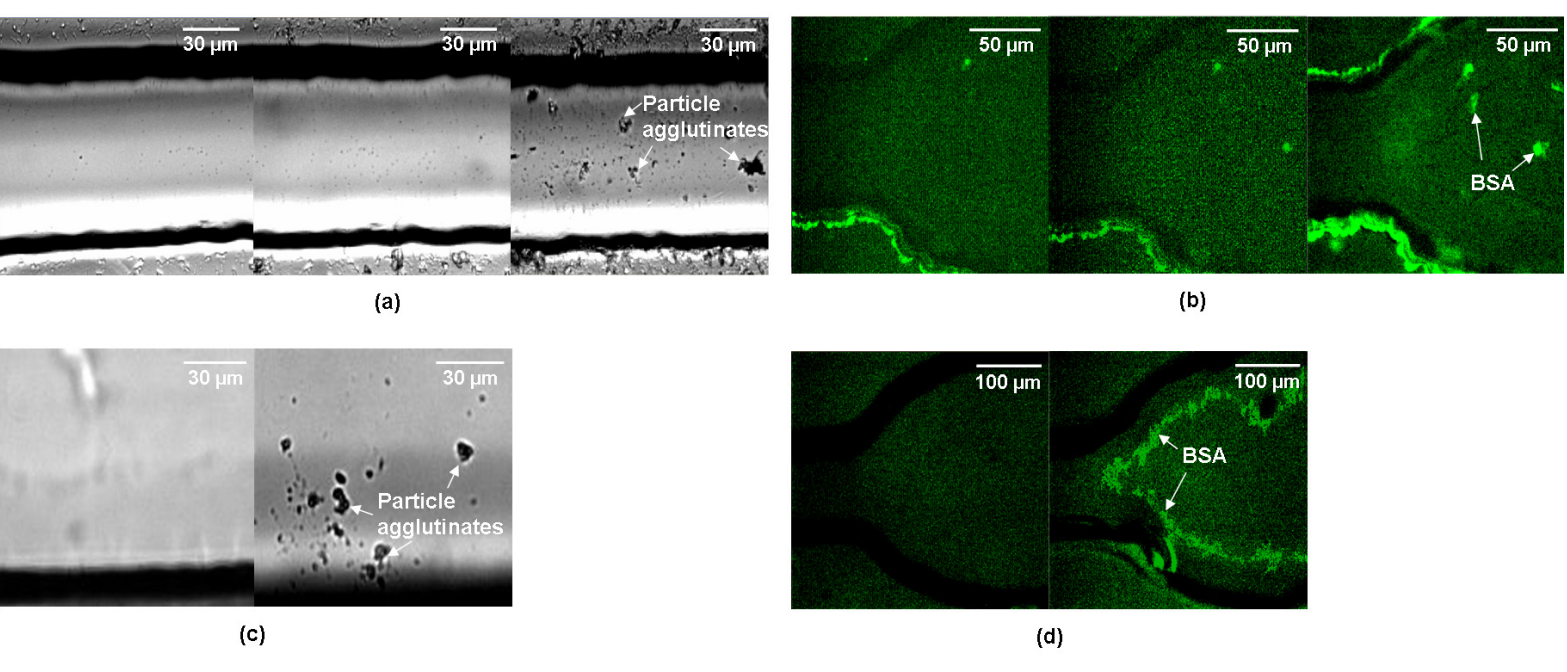
**Light and fluorescent microscopic images after repeated particle immunoassays (left) and BSA exposures (right)**. PEG (a and b) and oxygen-plasma-treated PDMS (c and d) microfluidic channels were used.

Fig. 2 shows representative, light and fluorescent microscopic images (out of three different sets of experiments) for latex immunoagglutination assays (a and c; taken 3-mm away from the Y-junction) and for injection of fluorescein-labelled BSA (b and d; taken at the Y-junction) after one, four, and ten uses of the same microfluidic channel. Immunoagglutination made the particles to form mostly triplets or larger clumps at high target concentrations (a few tens of μg ml^-1 ^and beyond), while mostly doublets at low target concentrations [17]. Therefore, we defined the microfluidic channel not reusable when those triplets and/or larger clumps could be observed in microscopic images after rinsing. We also defined the microfluidic channel not reusable when three or more bright fluorescent spots (of BSA) could be observed in microscopic images. The PEG microfluidic channels (a and b) were free of such particle agglutinates or BSA after four uses. Noticeable contaminations were observed only after ten uses. However, significant contaminations were observed for the "fresh" PDMS device after four uses. "Aged" PDMS device was a lot worse, showing significant fouling after the very first use (data not shown). This "aged" PDMS was virtually "plain" PDMS, since the hydrophobic recovery gained by oxygen plasma treatment fades away within 48 hours, known as hydrophobic recovery [18]. The actual number of reusable time may be much higher for PEG microchannel, since our target concentration (1 mg ml^-1^) was relatively higher than those of typical biological assays (in μg ml^-1 ^to ng ml^-1 ^scales).

### Contact angle

**Figure 3:**
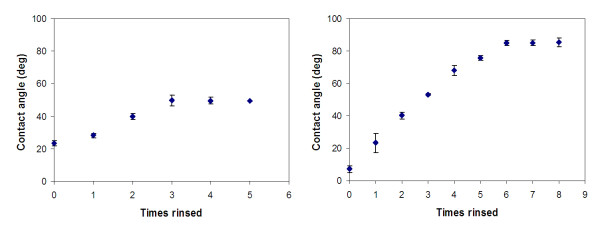
**Contact angles of PEG (left) and oxygen-plasma-treated PDMS (right) substrates upon repeated rinses**. 5 μl water drops were used. Error bars are standard deviation.

The water contact angles on PEG surfaces were obtained against the number of rinsing performed (Fig. 3). PEG surfaces initially showed very low contact angles (23°). This angle gradually increased and levelled off at 50° after three rinses and beyond. Meanwhile, the contact angles on "fresh" PDMS started at 7° (no rinsing), rapidly increased to 68° at four rinses, and levelled off at 85° after six rinses and beyond. The contact angle of ~60° seems to be a threshold in determining reusability of a microfluidic device for particle immunoassay or passive protein adsorption. As the contact angle on PEG surfaces stayed at 50° (i.e. below ~60°) even after 10 rinses (data not shown), the contaminations on a PEG microchannels after 10 rinses may be attributed to permanent protein fouling on surface cracks and/or dust particles, not by surface hydrophobicity.

This threshold angle of 60° can be correlated to those on protein films. Water contact angles on salt-free, dried protein films were measured as: 75 ± 1° for bovine serum albumin, 67 ± 1° for bovine hemoglobin, and 47 ± 1° for hen egg white lysozyme. These contact angles are comparable to our threshold angle of 60°. Once the water contact angle of microchannel surface exceeds those of proteins, hydrophobic-interactions-induced protein adsorption will be preferred on microchannel surface that will permanently foul the surface [19,20].

## Conclusion

Through this work, we reported that PEG-structured microchannel was not only protein fouling-resistant but also reused repeatedly. We hope that this device can be installed at a permanent location to perform unmanned bio-assays, eliminating the need for device replacement. More detailed biocompatibility studies should be followed for the other types of bio-assays and with various target biomolecules.

## Methods

### Particles and target proteins

In order to perform particle immunoassays, antibodies were conjugated to microparticles by physical adsorption as described previously [17]. Briefly, 1 ml of 0.02% w/v, 0.92 μm highly carboxylated polystyrene particles (parking area = 10.3 Å^2 ^per carboxyl surface group; Bangs Laboratories, Fishers, Indiana, USA) were mixed with 1 ml of 1.023 μg ml^-1^, anti-mouse immunoglobulin G (anti-mIgG; catalog number M8642, Sigma-Aldrich Co, St. Louis, Missouri, USA) solution, followed by centrifuging and resuspension (the whole cycle was repeated twice) to eliminate the free antibodies. The surface coverage of the antibodies on particle surface is approximately 33%, which is appropriate in maximizing particle immunoagglutination [21]. Target protein was mouse immunoglobulin G (mIgG; catalog number I5381, Sigma-Aldrich). For a comparison purpose, 1 mg ml^-1 ^fluorescein-labelled bovine serum albumin solution (with fluorescein isothiocyanate; FITC-BSA; catalog number A9771, Sigma-Aldrich) was used to monitor the protein-fouling behaviour within the PEG microfluidic channel. All dilutions were made with 10 mM phosphate buffered saline (PBS; pH 7.4; Sigma-Aldrich).

### Fabrication of PEG microfluidic channels

**Figure 4:**
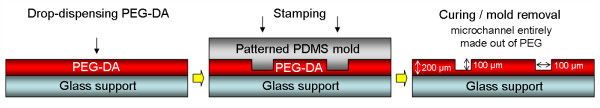
**Fabrication procedure of a Y-shaped PEG microfluidic channel**.

Fig. 4 (left) shows the layout of a Y-shape PEG microfluidic channel. PEG microfluidic channels were fabricated by drop-dispensing polyethylene glycol diacrylate (PEG-DA) with 1% w/v UV initiator (2,2-dimethoxy-2-phenylacetophenone) on a glass slide. The glass slide was pre-modified with adhesion promoter (acrylic acid dissolved in propylene glycol monomethyl ether acetate; 10 vol.%) [16]. This glass slide merely serves as a support for PEG-DA. A stamp made by the popular PDMS molding technique [22] was used to transfer a pattern onto a PEG-DA substrate as shown in Fig. 4 (right). As the PDMS mold did not make a contact with the glass support, microchannels made entirely out of PEG-DA could be fabricated. Microchannels were 100 μm wide and 100 μm deep. The PEG-DA layer on a glass support was 200 μm thick. A removable adhesive tape (Scotch^®^, 3 M, St. Paul, Minnesota, USA) was used as a cover for PEG microfluidic channel as shown in Fig. 4 (left). This tape cover could be discarded after each assay.

### Image analysis of microfluidic channels

Particle suspensions and/or protein solutions were introduced to a PEG microfluidic device via capillary action from the liquid droplets (3 μl each) sitting on the inlets, as shown in Fig. 1. Five minutes after introducing the solutions, an adhesive tape was removed and the microfluidic channel was rinsed with deionized water, followed by observation of the inner surfaces of a microfluidic channel with an inverted, light or fluorescent microscope (Nikon Instruments, Tokyo, Japan) (Fig. 1). This procedure was repeated until visually identifiable particle agglutinates (i.e. triplets or larger clumps) or BSA (i.e. three or more bright fluorescent spots) could not be removed from the microfluidic channel by rinsing. Additionally, we used PDMS microfluidic channels (i.e. the most popular device) as negative controls, with the same layout and dimensions of microchannels. Both "fresh" oxygen-plasma-treated PDMS (water contact angle < 10°) and "aged" PDMS (two days of incubation at room temperature after oxygen plasma treatment; water contact angle ~90°) were tested. The fabrication procedure of PDMS microfluidic device can be found in previous publications [17,23].

### Contact angle

A contact angle/surface tension analyzer (FTÅ200, First Ten Ångstroms, Portmouth, Virginia, USA) was used to measure the contact angles on the PEG surfaces with 5 μl sessile drops of deionized water. To simulate water rinsing of a microfluidic channel, the PEG surfaces were rinsed with deionized water after each contact angle measurement. Sessile drops were placed on the surfaces for 2 min, the same as the liquid exposure time of PEG (and PDMS) microfluidic channels. A single data point was averaged from three different measurements of contact angle on PEG (and PDMS) surfaces. Substrates were thoroughly dried with nitrogen gas prior to contact angle measurement.

Water contact angles on salt-free, dried protein films were also measured using the same instrument. Bovine serum albumin (catalog number P-7656, essentially salt-free, Sigma, St. Louis, Missouri, USA), bovine hemoglobin (catalog number H-9891, essentially salt-free, dimethylated and primarily methemoglobin, Sigma), and hen egg white lysozyme (salt-free, catalog number 10 837 059 001, Roche, Germany) were used as model proteins. 15 μl of 1 mg ml^-1 ^albumin, haemoglobin or lysozyme solution was deposited on a precleaned glass microscope slide (catalog number 12–552, Fisher Scientific, Pittsburgh, Philadelphia, USA), and stored in a nitrogen-purged desiccator for more than a week. 5 μl droplets of deionized water (from Millipore's Simplicity, resistivity > 18 MΩ cm) were automatically dispensed by the same contact angle analyzer, and deposited on the protein films. Contact angles were measured right after a droplet stops vibrating and forms a perfect spherical shape. This was normally achieved within 33 ms (images were captured every 33 ms). Sometimes the contact angle kept decreasing significantly over time, probably due to the absorption of solvents into the films. We simply eliminated such data from our experimental set.

## Competing interests

The authors declare that they have no competing interests.

## Authors' contributions

JHH and JYY designed/analyzed the experiments and wrote the manuscript. JHH fabricated the PEG and PDMS microchannels and performed particle immunoassays. JYY conceived the original idea and measured water contact angles of protein films.
